# Connect Coordination Victoria and the Establishment of a State‐Wide Network of Family Carer Mental Health and Well‐being Connect Centres: Family Carer Lived Experience Leadership Through Online Community Co‐Design, Communities of Practice and Power Sharing

**DOI:** 10.1111/hex.70803

**Published:** 2026-08-19

**Authors:** Jessica Phillips, Lisa Casaceli, Clare Fleming, Jennifer Bite, Caroline Walters, Joanna Tilkeridis, Marie Piu, Melissa Petrakis

**Affiliations:** ^1^ Tandem Melbourne Australia; ^2^ School of Social Work Monash University Melbourne Australia; ^3^ Mental Health Service St Vincent's Hospital Melbourne Australia

**Keywords:** caregivers, Communities of Practice, families, leadership, lived experience, mental health, online community, power sharing, reflexive thematic analysis, values‐led

## Abstract

**Background:**

This paper builds upon previous research in which we systematically identified the coordinating values of Connect Coordination Victoria: a non‐government, family carer‐led coordination body overseeing a network of tailored services for mental health family carers in Victoria, Australia. The four core values we identified were: connection, responsiveness, consistency and flexibility. Wanting to see if these values continued across additional data, led us to a second iteration of our initial findings. In this paper, we describe secondary findings related to Connect Coordination Victoria's values with special attention to how power was acknowledged and addressed across their activities.

**Methods:**

The research team used document analysis and reflexive thematic analysis to systematically identify, code, and analyse *n* = 41 documents (17,630 words) that captured the coordination activities of Connect Coordination Victoria between November 2023 and September 2025. We describe our analysis of documents related to two Connect Coordination Victoria‐led projects: an online community co‐design project and Communities of Practice. We sourced documents using a best‐practice, systematic process that evaluated each document according to six factors: relevance, authenticity, credibility, accuracy, representativeness and completeness. 10,365 words were included for analysis in this study.

**Results:**

We determined *n* = 3 core coordinating values of Connect Coordination Victoria continued across the data. These values were: connection, consistency and flexibility. We also found that identifying and addressing power dynamics underscored the enactment of Connect Coordination Victoria's values.

**Discussion:**

Our research demonstrated that Connect Coordination Victoria created a specific power dynamic between themselves as coordinators and the family carer‐led Connect centre workforce. This power dynamic privileged values of connection, consistency and flexibility. Connect Coordination Victoria's family carer lived experience leadership represents an important case study for the success of values‐led lived experience coordination of tailored services for family carers in mental health.

**Conclusion:**

This research lends support to the need redistribute power across mental health systems so that those with family carer lived experience can step into leadership roles that enable them to shape the services and systems that directly impact them and those they care for.

**Patient or Public Contribution:**

This research was developed, designed, conducted, and written by researchers with family carer lived experience. The secondary data we analysed related to the family carer‐led coordination of a tailored service for mental health family carers. All authors hold a lived experience of caring for and/or supporting people experiencing mental health challenges. No generative AI was used at any stage of this research project.

## 1 Introduction

Globally, mental health challenges are experienced by an estimated 1.095 billion people [1]. Despite the prevalence of mental health challenges, many people remain ‘severely underserved’ by mental health systems due to worldwide gaps in ‘information, governance, resources, and services’ [1, p. 32]. One cohort of people impacted by mental health challenges but not accounted for in these figures are the friends, parents, partners, family, carers, and kin who provide unpaid support to people experiencing mental ill health.

In Australia, approximately 971,000 people provide unpaid, informal care to those experiencing mental health challenges [2, p. 872]. These figures are likely conservative because many friends and family members do not identify with the term *carer*. One Australian study of the experiences of young carers found that young people do not see themselves as ‘carers’ but as ‘the responsible one’ where they can feel it ‘is simply their responsibility’ to care for family members [3, p. 16]. While ‘carer’ may be a policy‐based term that does not reflect the characteristics of many ‘caring’ relationships, in our study, we use the term ‘family carer’ to include family of origin and family of choice who provide unpaid, informal support to people experiencing mental health challenges [4, 5, 6, 7].

Despite increased government spending on mental health in Australia ($12.2 billion in 2021–2022, up 3% from 2017 to 2018), family carers, like many millions of consumers, are underserved by mental health services [8, p. iv]. The recent Royal Commission into Victoria's Mental Health System (RCVMHS) (2019) received over 12,500 contributions from consumers, family carers, and mental health workers via focus groups, personal statements, consultations, and direct testimonies [9, p. 14]. These contributions revealed that in elevating other government spending priorities above mental health coupled with an ingrained ‘individualistic approach to treatment,’ the Victorian mental health system can overlook the vital role played by family carers in aiding the recovery of consumers [10, p. 72]. These state‐specific findings are congruent with international research into the experiences of mental health family carers who frequently report feeling ‘disrespected,’ ‘tacked on,’ ‘let down,’ ‘powerless,’ and in some cases ‘abandoned’ by underfunded mental health services struggling under the weight of increasing demand [11, p. 2756–2758; 12, 13, p. 194; 14].

### 1.1 Mental Health and Wellbeing Connect Centres

**Figure 1:**
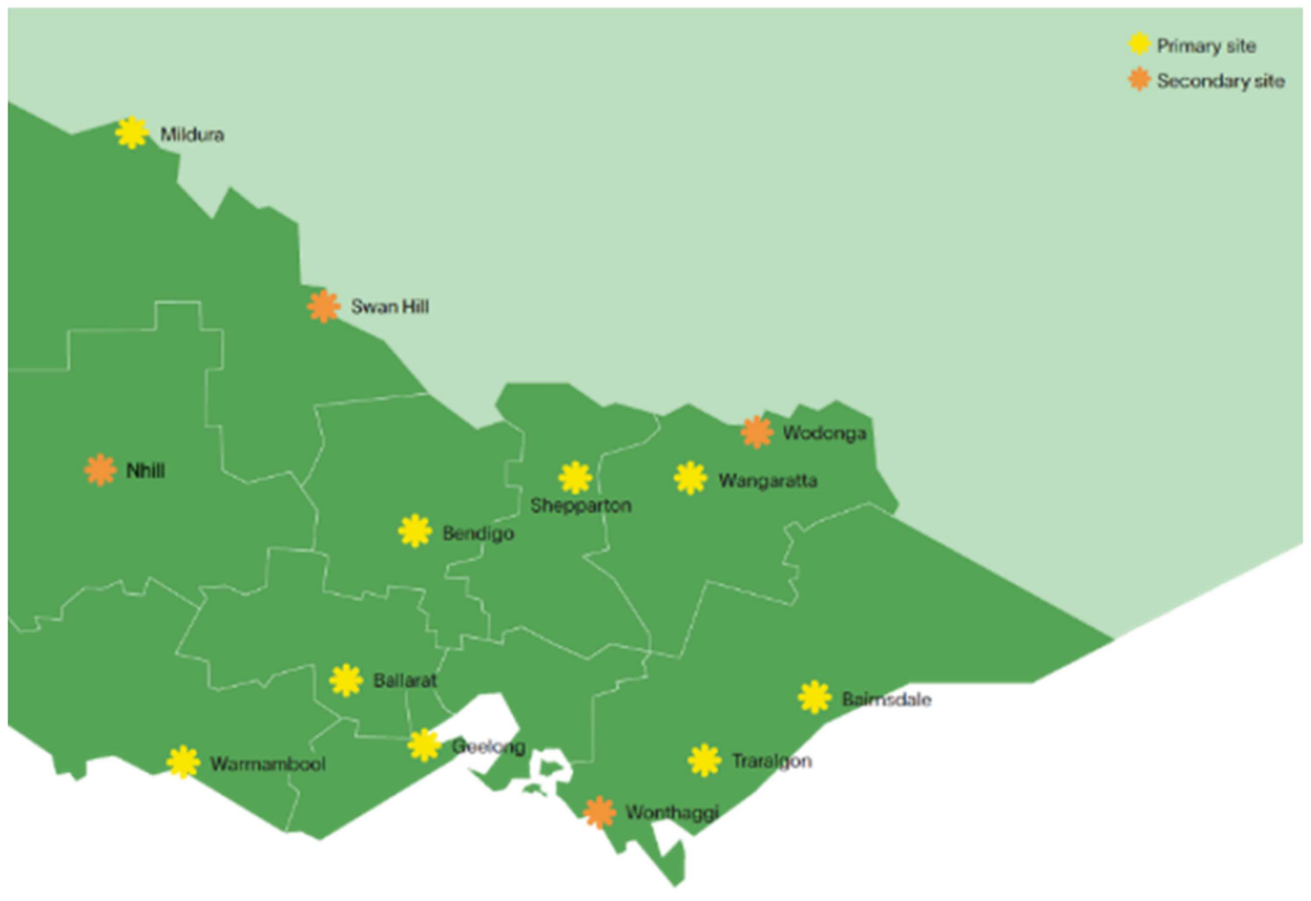
Map of regional connect centres (Tandem, 2025).

**Figure 2:**
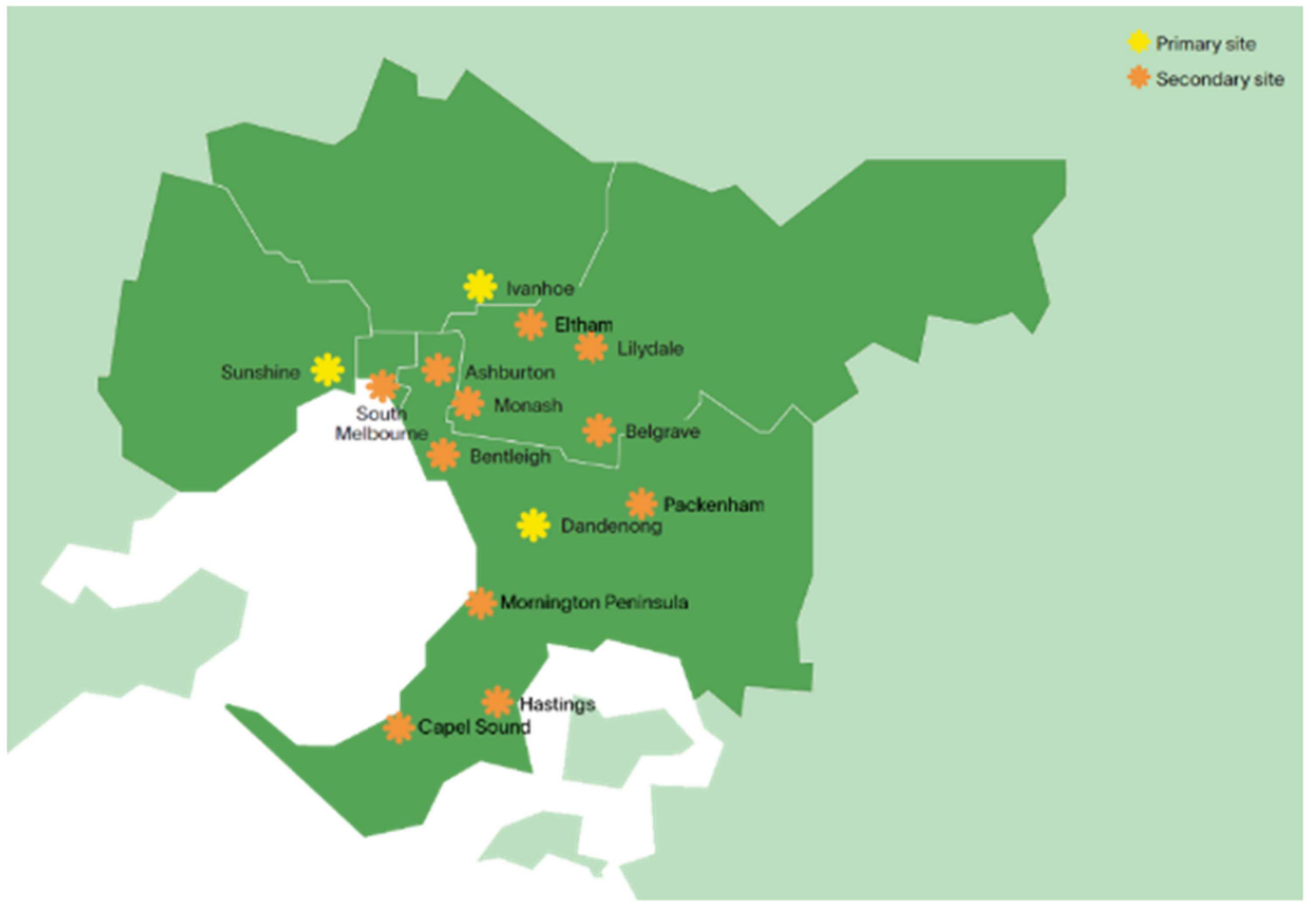
Map of metro connect centre locations statewide (Tandem, 2025).

**Figure 3:**
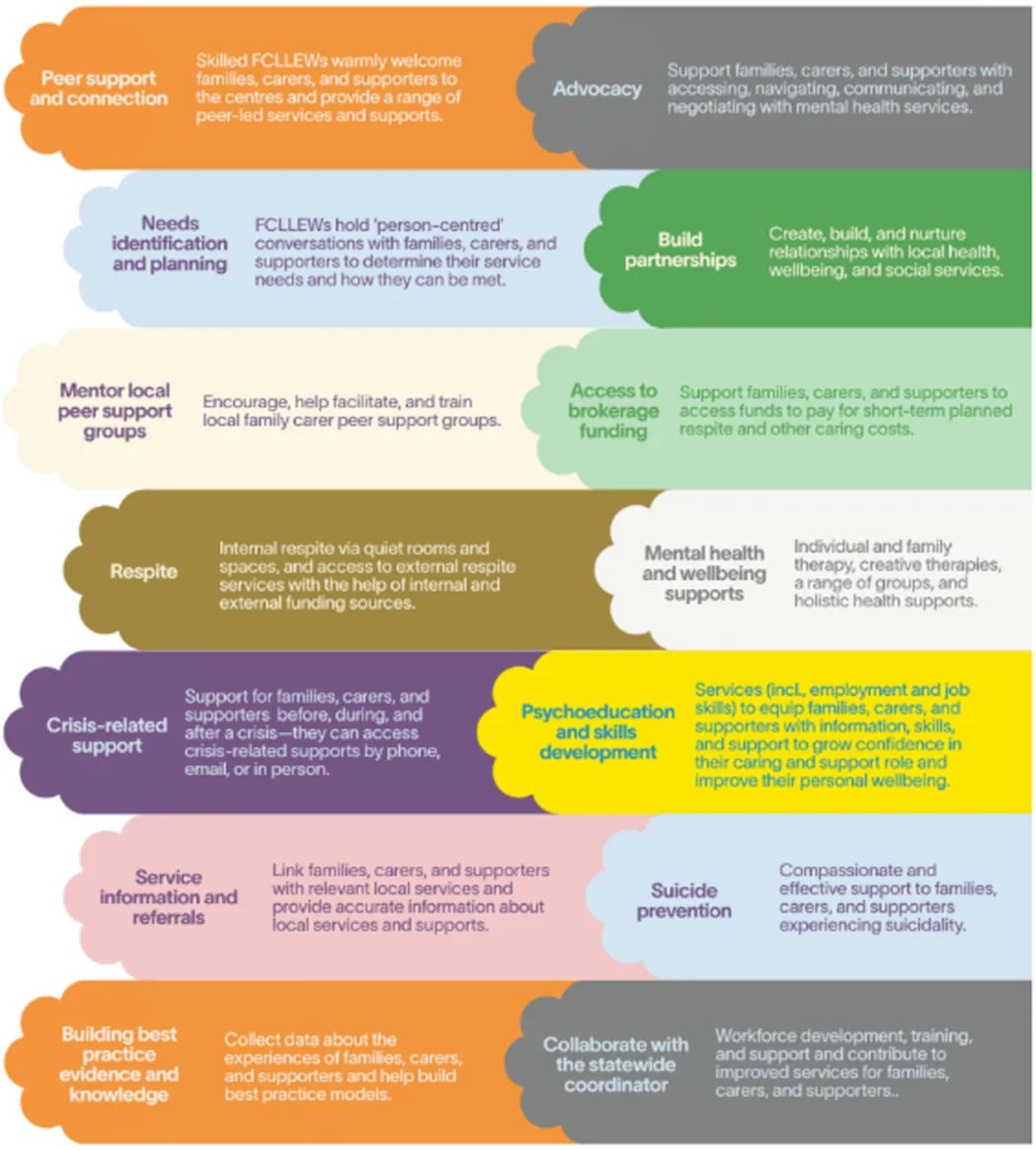
Core service features offered by Connect centres (adapted from Tandem, 2023, Service Development Framework, p. 5–6).

To address the need for tailored services to empower and support family carers, the RCVMHS recommended the state‐wide establishment of a network of dedicated family carer peer‐led services. These services, known as the Mental Health and Wellbeing Connect centres (Connect centres) were established following the RCVMHS and now operate out of eight regions across the State of Victoria. Maps and core service features are illustrated in Figures 1, 2 and 3 and additional information regarding their establishment can be found in our companion paper [15].

### 1.2 The Role of Connect Coordination Victoria (CCV) and Tandem

The Victorian Government initiated a tender process in 2023 to identify an organisation to provide statewide support and coordination to the Connect centres. Tandem secured this role in October 2023, six months after the Connect centres opened.

Tandem was successful in obtaining the coordination role because they are the trusted Victorian peak body for mental health family carers and have advocated for the voices of family carers for more than 30 years. Tandem played a central role in establishing the Connect centres by developing the statewide Connect centre service delivery model after the RCVMHS through sustained advocacy and co‐design efforts alongside family carers. Tandem is led and staffed by many people in designated family carer lived experience roles. This family carer lived experience lens throughout Tandem extends to CCV and as a result, the voices of mental health family carers are embedded across the entire Connect centre service delivery model. The Connect centre model is uniquely positioned to offer family carer peer‐to‐peer services under the guidance of the Tandem‐run CCV: a non‐government coordinating body that leads from and with their family carer lived experience.

### 1.3 Aim

In our companion paper, we identified the coordinating values of CCV using reflexive thematic analysis and document analysis methods [15]. We analysed secondary data concerned with the activities of CCV across two core projects (Educational Forums and a Mini‐Showcase event). From this analysis, we identified four core values that underpinned CCV's coordination: connection, consistency, responsiveness, and flexibility [15]. Seeing these values and themes continuing in the data, led us to a second iteration of our initial findings. Our primary aim in this study was to understand how/if CCV's values were expressed across two different CCV‐led projects: Communities of Practice (CoP) and the co‐design of an online community. Here, we build upon our earlier secondary data analysis to describe additional findings related to CCV's values.

Reviewing the continuation of CCV's values was important because values are central to the practice of the family carer lived experience workforce discipline [15, 16]. In this study, we wanted to explore how CCV's values were expressed in nuanced ways over time to understand how coordinators can hold and demonstrate their values when supporting the delivery of a mental health service by family carers for family carers.

The research question underpinning this inquiry was:

***How does a family carer‐led coordination body enact their values through concrete activities?***

To explore this research question, we chose to analyse secondary data related to the online community co‐design project and CoPs (see Tables 2 and 3) because these projects described sufficiently different activities to the Mini‐Showcase event and Educational Forums described in our companion paper [15]. We chose to analyse secondary data because it was a readily available data source that lowered the resources (both time and money) required by our small not‐for‐profit research team to complete this research. We hope the findings we discuss foreground for others what can be achieved by analysing readily available data. Our analysis of secondary data does not stand as an alternative to field work and interviews, but as a compliment and an insightful preliminary research stage within the scope of a larger, ongoing inquiry.

### 1.4 Co‐Design, Online Communities and Communities of Practice

Co‐design and/or ‘co‐production’ are terms used widely across mental health research and service design [17, 18, 19, 20, 21, 22]. Despite the growing ubiquity of these terms, and the muddy, often ambiguous definitions applied to related ‘co’ words, at their core, co‐design methods seek ‘leadership’ from people with an experience of the problem or service ‘from the outset’ [19, 20, p. 2]. Co‐design approaches recognise that early and ongoing involvement of people with a lived experience of the problem or service must happen to design relevant services and supports for them [20]. CCV commenced the co‐design, development, and initial implementation of the online community we analysed (now known as *Connect Community*) by establishing a working group of *n* = 19 Connect centre staff from across the newly established Connect centres. The co‐design process commenced in 2023 when the Connect centres—staffed by a brand‐new workforce—were just forming. At this early stage of development, the Connect centre staff may have lacked complete clarity around how the Connect Community could support their direct practice with family carers because they were still trying to understand the scope of their roles in the brand new Connect centres.

**Figure 4:**
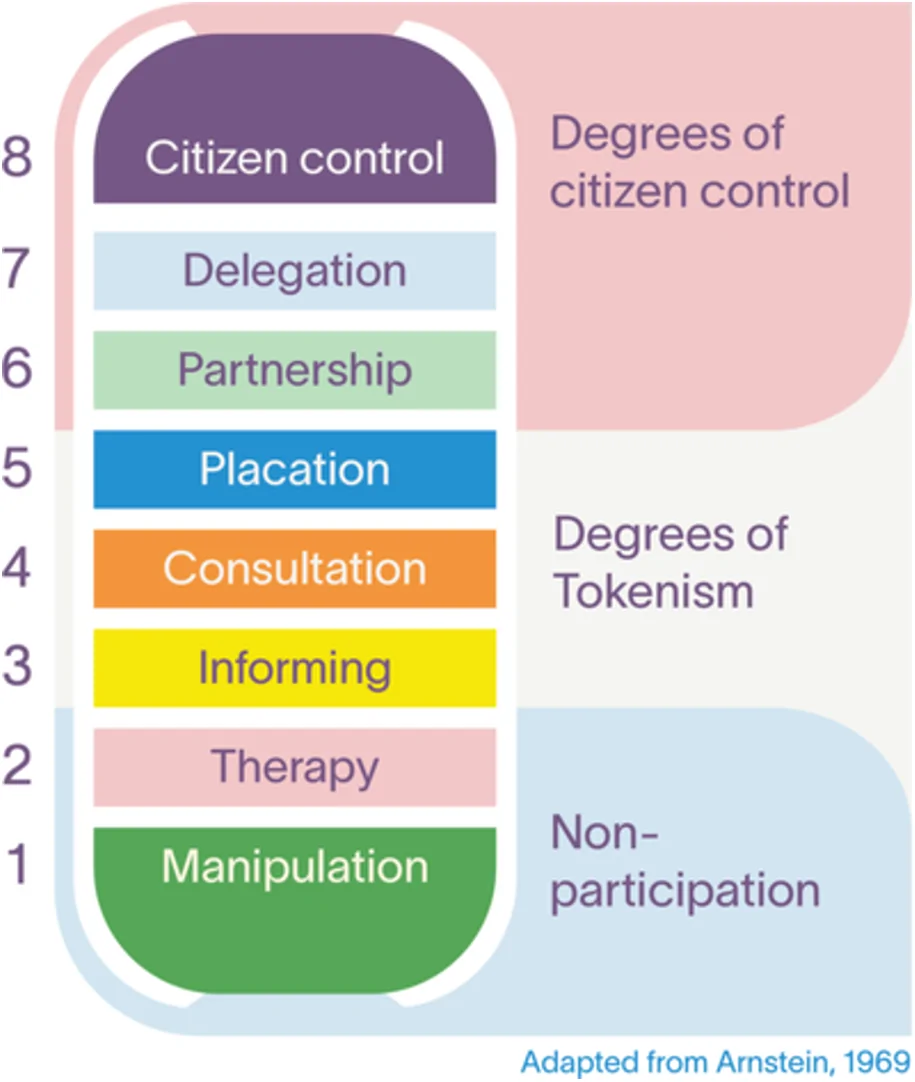
Arnstein's ladder of citizen participation.

‘Co‐production’ and co‐design activities are typically exercises in identifying, negotiating and redistributing power. And while the term ‘co‐production’ is often traced to Ostrom et al. [23], it shares ties with Arnstein's earlier 1969 work on citizen participation. Arnstein's ladder, (see Figure 4) shows eight levels of participation which correspond with people's power in influencing decisions about services, programs, and supports that directly affect them. Arnstein's work is, not coincidentally, the resource adopted by the Commission in the Final Report of the RCVMHS (2021). Arnstein's work served as the template for the Commission in thinking about how to redistribute power so that people with lived experience can co‐lead the services, initiatives, and projects that directly affect them [10, p. 19–20].

As outlined, in this inquiry, we analysed documents related to the CCV‐led Communities of Practice for Connect centre workers (see Tables 2 and 3). CoPs defined by the anthropologist Etienne Wenger as ‘groups of people who share a concern or a passion for something they do and learn how to do it better as they interact regularly’ can serve as a kind of ‘home base’ for people with a shared identity [24, p. 241; 25]. CoPs are ‘the basic building blocks of a social learning system’ where people can mutually define and redefine their identities and what it means to be competent while forging and maintaining strong relationships of belonging to their discipline and to one another [24]. With these qualities in mind, the redistribution of power seems inherent to CoPs because they operate outside of the hierarchical and academic institutions that have, historically, governed how and what members of a discipline learn. CoPs, in a general sense, are not a replacement for formal learning; they are ‘communal’ spaces where learning occurs through ‘mutual engagement’ and the sharing of experiences, ‘tools, stories, [and styles]’ [24, p. 229].

All of these ‘ideal’ qualities of CoPs suggest they are well suited as a learning platform for mental health family carer lived experience workers in the Connect centres because they encourage the mutual sharing of lived and learned wisdom to build the capacity of everyone within the group [24, p. 230]. The mutual sharing of lived and learned wisdom has long been a practice principle of lived experience peer support via Sherry Mead's Intentional Peer Support (IPS) model, and ‘mutuality’ has recently been defined as a core practice principle of the mental health family carer discipline [16, 26]. CoPs and family carer lived experience work seem well matched as they share the same values‐based practice approach to learning. The reciprocity, trust, give and take, and learning with one another that takes place within CoPs has power at heart because this social learning system acknowledges that each member has the power and right to contribute knowledge to the system.

In this research study, our interest lay with if and how the online community co‐design project and the CCV‐led CoPs enacted CCV's core values and how they related to and expressed power given the connection both co‐design and CoPs share with negotiating power dynamics.

## 2 Materials and Methods

### 2.1 Research Design

As detailed in our companion paper, we employed two qualitative research methodologies: document analysis (DA) and reflexive thematic analysis (RTA) to systematically identify the core values informing CCV's coordination of the Connect centres [15, 27]. We described both methods in detail in our companion paper, including our rationale for each [15]. We offer a truncated description of our methods below. As this research was family carer led, those with family carer lived experience were involved across all stages of the research from conceptualisation, analysis, theme generation, write up, to the final submission of this paper.

Our approach to DA and RTA involved four main phases: selection; coding and extracting key quotes; generating themes; and member reflections. These four phases aligned with Bowen [28] and reflect the core phases of RTA described by Braun and Clarke [29], with the addition of a member reflection phase, adapted from Tracy [30]. Our primary researcher, JP, was best positioned to lead the data analysis for this research because she commenced at Tandem in May 2025 and had no previous involvement with CCV.

### 2.2 Data Collection

Phase 1: Selecting and identifying documents for analysis

The first phase involved systematically identifying and selecting documents for analysis according to six factors (adapted from [28] and [31]). These factors, illustrated in Table 1, align with those adopted in our companion paper.

**Table 1:** Selecting and identifying documents for analysis.

Factor	Questions for identifying and selecting documents
Relevance	Do the documents relate to CCV and their role in coordinating the Connect centres? Were they produced between October 2023 and 30 August 2025?
Authenticity	Did a member of CCV/Tandem create the document? Can we determine the author/creator? Is the document an original, genuine source of information about CCV?
Credibility	Who created the document, and what is their role in CCV? Does the author hold a conflict of interest? Does the perspective of the article appear to represent some interests at the expense of others? What does the document aim to achieve? How many errors appear in the document?
Representativeness	Does the document reflect the nature and approach CCV took to coordinating the Connect centres (either implicitly or explicitly stated)? Does the document link with the other documents already sourced? Does the document add a new perspective on CCV not covered by other documents or does it offer support to documents already collected?
Meaning	How significant is the content of the document? Does the content read well—understandable, clear, and fit for analysis? Does the document have literal (face‐value, semantic) and/or interpretative (deeper, latent) meaning?
Completeness	Does the document cover CCV in a complete sense, or offer a small window into their coordination role?

For our analysis, we completed the story we began writing in our companion paper by drawing on complementary yet different secondary data from the CoPs, and online community co‐design project (see Tables 2 and 3). Through group discussion, we determined that 10,000 words for analysis would enable us to substantiate meaningful and significant claims about CCV's activities and their values. 10,000 words also set the words for analysis at a manageable limit to enable our early career researcher to complete the project within six months to accord with academic mentoring.

**Table 2:** Online community co‐design project.

Document type	Word count
Meeting minutes: Second co‐design group 28/11/2023	413
Meeting minutes: Third co‐design group 5/12/2023	414
Meeting minutes: Fourth co‐design group 10/01/2024	1277
Meeting minutes: Fifth co‐design group 22/02/2024	1132
Meeting minutes: Sixth co‐design group	580
Ways of working document	151
Getting online and navigating the online Connect Community: Introductory document	775
Total words	4742

**Table 3:** Communities of Practice (CoPs).

Document type	Word count
Connect centre CoP all staff purpose document	472
Connect centre CoP all staff participation agreement	251
Draft survey for CoP all staff	376
Connect centre CoP counsellors and clinicians purpose document	506
Connect Centre CoP counsellors and clinicians' participation agreement	251
Community of practice topics feedback document	122
Connect centres CoP all staff survey results July 2025	1105
Connect centres CoP counsellors and clinicians' survey results July 2025	301
CoP September 2025 report (draft)	2239
Total words	5623

### 2.3 Data Analysis

#### 2.3.1 Phase 2: Coding and Extracting Key Quotes

The second phase involved coding and analysing the selected documents. To code, our primary researcher, JP, uploaded the selected documents to the qualitative cloud‐based software, Quirkos. She then undertook three core actions for each document. These three actions, adapted from Bowen [28] and integrating RTA phases included:

1. Skimming the selected documents to identify ‘meaningful and relevant passages of text and data’ [28, p. 32].
2. Rereading the documents, examining their contents in detail, extracting key quotes, and creating a set of initial codes.
3. Refining the codes through subjective interpretation.

Our research adopted what Braun and Clarke refer to as ‘Big Q,’ qualitative research that takes place within a ‘non‐positivist’ framework [27, p. 1]. Positivist frameworks begin from the assumption that ‘a single tangible reality exists’ and knowledge about this reality must develop objectively without the ‘values [or biases] of the researcher or participants influencing its development’ [32, p. 691]. In contrast, our research embraced the subjectivity our primary researcher who as the primary coder, engaged in reflexive practice. She journalled while coding and discussed her observations, challenges, and biases regularly with M. Petrakis. In the coding phase, we sought a subjective interpretation of the data with the aim of understanding how CCV's values continued across the online community co‐design project and CoPs.

#### 2.3.2 Phase 3: Generating Themes

In this phase, we explored shared and united meaning across the data with the aim of telling an ‘interpretative story’ about the continuity of CCV's values across different CCV‐led activities [27, p. 2]. From her coding in Phase 2, our primary researcher, JP, generated initial themes, which she cross‐checked with the original values. She then met with M. Petrakis to discuss her interpretations. Following these discussions, our primary author refined her themes and engaged her family carer lived experience co‐authors in one round of member reflections to complete the full draft of the manuscript.

#### 2.3.3 Phase 4: Member Reflections

In phase 4, our primary researcher engaged in one round of member reflections alongside two of her family carer lived experience co‐authors (LC and CF) who worked within CCV. The purpose of these ‘member reflections’ were not to achieve a consensus interpretation of the data, but to invite the perspectives of those with family carer lived experience in leadership roles (CCV) to provide their insights *during* the analysis phase. Via our member reflections, ‘credibility’ moved beyond trying to verify the results and extended to enacting credibility as a ‘process’—an opportunity for further collaboration and elaboration on the findings so far [30]. In the case of a family carer‐led research project, credibility involved active collaboration, sharing power, inviting endorsement, affirmation, and critique. Each member asked and responded to questions that moved the research forward in so far as our primary author could review, revise, and incorporate their fresh insights into the analysis. Member reflections had the positive side effect of generating goodwill between co‐authors because they were not tokenistic: the two co‐authors had real power to shape the final analysis [33]. And they did—the insights from these member reflections expanded and improved the resulting analysis without compromising the methodological rigour and subjective interpretations of our primary researcher in the previous phases. In this phase, an additional document (CoP report, 2025, draft) came to the attention of the primary researcher. Her co‐authors urged this document to be incorporated into the analysis given the restrictions on analysing confidential documents related to the content of the CoP sessions. This document was subsequently coded and analysed by the primary researcher, JP, and her findings were combined into the resulting analysis. The report was drafted in September 2025 and is the only item drafted after August 2025 to be included for analysis.

## 3 Results

### 3.1 Overview

The three themes we developed continued the core values embodied by CCV in coordinating two primary projects: the online community co‐design project and the CoPs. We found that all three values were informed by attention to power dynamics. The *n* = 3 values we observed continuing through the data included: connection, consistency, and flexibility. The three themes we developed from these values were: Sharing Power Seeds Connection, Consistent Rituals Keep Power in View, and Flexibility Softens Power.

Tables 2 and 3 summarise the documents we drew from for our analysis including the document type and the word count. We included 10,365 words for analysis in the current study. As CCV is family carer‐led, the documents we analyse were developed by those with family carer lived experience and relate to their coordination of a tailored service by and for family carers in mental health.

### 3.2 Summary of CCV Documents Included for Analysis

#### 3.2.1 Theme 1: Sharing Power Seeds Connection

The primary theme we developed through this research project reflects the central value of CCV's coordination: *connection*. Here, we build upon our previous findings by focusing on the online community co‐design project and CoPs to show how this value continued across these two projects. In discussing connection as CCV's core coordinating value, we link this value directly with our corresponding finding about CCV's ongoing identification, acknowledgement, and address of power between themselves and the Connect centre workforce. In developing this theme, we first describe the online community co‐design project.

The introductory document for the online community, now known as *Connect Community*, stated that the site is ‘a‐[n] [online] platform for Connect centre staff’ that aims to ‘facilitate ease of communication and collaboration between centres.’ Through our member reflection phase, as detailed in our methods above, CCV staff informed JP that the Connect Community now serves as a central repository for training material and resources that Connect centre staff can draw from to support their direct practice with family carers in the Connect centres. CCV clarified that through their coordination role, they now provide technical and moderation support via the lens of their family carer lived experience and expertise to keep Connect centre staff engaged. Our analysis of the Connect Community remains limited to the initial design of the platform, not current use.

As detailed in the original Call for Funding Submission document (CFS), the Department of Health (DoH) specified that the successful tender for the Statewide Coordinator role (CCV was appointed in 2023) needed to develop and implement an online community as part of their workplan. In the CFS document, DoH stated that organisations applying for the statewide coordination role would need to:

establish an online presence [for the Connects] that can provide information, shared resources, digital education products, and share communications between the Centres to support service provision [34, p. 13].

In meeting minutes from November 2023, CCV informed the online community working group members that ‘because the Department [DoH] is funding the process, there are parameters and deadlines which prevent the process [of designing the community together] from being pure co‐design.’ In the case of the online community co‐design, we saw CCV enact connection as a value by recognising that bringing Connect centre workers together through a co‐design process needed to happen even if a ‘co‐design’ ideal was and could only be imperfectly achieved due to workplan constraints and the newness of the Connect centre service delivery model.

In their approach to co‐designing the online community with Connect centre staff, CCV actively acknowledged, explored, and addressed power with the *n* = 19 working group members. For instance, prior to beginning the work of designing the online community together, CCV established a ‘Ways of Working’ document with the group. The Ways of Working document, a documented process used consistently across other CCV‐coordinated projects, served as an agreement for how the group would respectfully collaborate on the design of the online community. This document demonstrates that CCV sought to identify and address power differences and to share decision‐making power before any work began. For example, the document invited members to commit to providing ‘everyone with an opportunity to be heard’ and to ‘create[e] an environment where there is equal power sharing.’ The document was amended twice after suggestions from the group on 14 November 2023 to add an explicit reference to equal power sharing, and again on 28 November 2023 when the group suggested an amendment to add ‘inclusive’ to the working together document to create a ‘safe and confidential environment’. On 28 November 2023, the group agreed to use the document as a starting point for ways of collaborating on the design of the online community with the caveat that anyone, at any time, could amend the document.

Throughout the co‐design process, CCV undertook subtle gestures which added into a larger acknowledgement and address of power. For example, CCV invited working group members to contribute names for the online community, to which all members then voted to determine the preferred name. Members present at the meeting on 28 November 2023 submitted their ideas, but a decision was not made until everyone had the chance to vote. This appears like a non‐trivial action, but to delay decision making until all members had a chance to vote within the context of a time‐limited project, demonstrated how CCV assigned the same level of importance to connection and inclusion, shared‐decision making, shared power, and therefore shared ownership of the online community as to meeting deadlines for completion.

Another small gesture from this same meeting involved an end‐of‐meeting reflection whereby the chair acknowledged the difficulty in the task they were working towards. She acknowledged, as documented in the meeting minutes from 28 November, that ‘all online communities take time to get started’ and ‘at this stage, we're all trying to work it out.’ While this gesture appears subtle, it demonstrated a commitment to including the Connect centre working group members in decisions that impacted them, while reflecting a willingness on CCV's part to coordinate this project with the humility that concedes power in knowing how best to design an online community from the ground up.

#### 3.2.2 Theme 2: Consistent Rituals Keep Power in View

Above, when discussing the online community co‐design project, we spoke to the function of Ways of Working documents. While repeated less often than Recognitions of Family Carer Lived and Living Experience across CCV‐coordinated projects—across 50% of the projects analysed for this research as Table 4 demonstrates—we noted above that Ways of Working documents mattered in the context of CCV's coordination. They mattered because they existed to address the impact of power differences on effective collaboration and connection by inviting all members of the online community working group to collaboratively decide and agree to adhere to core principles that would create a safe work environment. Here, we build upon our discussion of Ways of Working documents and situate them as a tangible, repeatable instance of two core CCV values: connection and consistency. Below is an example of a Ways of Working document from the all‐staff CoP:

**Table 4:** Presence of Ways of Working documents across CCV‐coordinated projects.

Project	Presence of/ways of working documents (Y/N)
CoPs	Yes. Participation agreements formed for both all‐staff and counsellor and clinician sessions.
Online community co‐design	Yes, ways of working documents created in first co‐design meeting.

How we wish to work together to learn safely, and effectively, in a collaborative way for all who attend these sessions:

- Listen genuinely, carefully, and actively
- Practice empathy, compassion, and humility
- Be curious to learn from perspectives different to yours
- Ask questions, seek to understand, and encourage input from others who may feel less confident putting forward their views
- Confidentiality, what we say in the group stays in the group—only shared information will be in the agreement by the group e.g: Addressing gaps, areas requiring further support, topics, or themes to discuss and workshop
- We are all responsible for creating brave and safe spaces
- Have an open mind
- Raising hands to speak
- Writing in the chat to communicate if unable to raise hand
- Being respectful and mindful of how we receive feedback or provide opinions or feedback to one another
- Being culturally responsive and creating a space where all group members are comfortable to share if they want to

CCV invited members of the all‐staff CoP, to continue to shape ways of working together long after the initial Ways of Working document was forme*d 'by*‘*do[ing] a check‐in before commencing each session to ensure everyone has agreed to the above list or requires any additional changes*’ (italics original). The ongoing invitation to revisit these documents acknowledged CCV's power in so far as they could have chosen not to seek ongoing involvement from Connect centre staff. CCV therefore held themselves to account by regularly inviting Connect centre workers to challenge and amend how the CoPs worked. These regular check‐ins also served to distribute power across the group by formally and regularly recognising that everyone held the power to positively shape their work culture to create a safe, effective, and collaborative environment for all.

#### 3.2.3 Theme 3: Flexibility Softens Power

For this final theme, we focus on the framing and purpose of the CoPs. Due to the confidential nature of the sessions, we limited our analysis to CoP process and purpose documents.

The CoP Report document detailed that the CCV‐led Community of Practice (CoP) sessions were established with the goal of ‘support[ing] and connect[ing]’ Connect centre staff so that these staff can ‘continuous[ly]’ improve, learn, and uphold the best possible ‘service delivery model’ to ‘families, carers, and supporters accessing the Connect centres.’ Before the CoPs commenced in January 2025, CCV created a purpose document that defined a CoP in general terms alongside the explicit purpose of the CCV‐led CoPs. Borrowing from Wenger et al., CCV defined a CoP as, ‘[g]roups of people who share a concern, a set of problems, or a passion about a topic and who deepen their knowledge and expertise in this area by interacting on an ongoing basis.’ CCV also stated that the CoPs intended to ‘provid[e] an opportunity to collaborate, learn and create together through addressing group requests on themes or areas of practice that are of interest to the group to discuss and workshop;’ while also carving out dedicated time and space for ‘problem solving and sharing successes’ and ‘identifying different training and our skill building needs to support this work.’

From the outset, CCV distributed power across the workforce by inviting them to identify session topics for discussion. In a preliminary survey to determine preferences for all‐staff CoP sessions between January 2025 and August 2025, two Connect centres contributed responses (see Table 5). Seven of ten topics suggested by these workers were delivered across eight all‐staff sessions, which equates to 90% of the all‐staff sessions conducted during this period aligning with their initial preferences. These results are displayed in Table 5.

**Table 5:** Suggested CoP topics for all‐staff sessions.

Topic suggested by Connect centre staff	Date CCV dedicated a CoP session to that topic
Advocacy/self advocacy	March 2025
Dignity of risk	Not covered
Relational recovery	Not covered
Lived experience to lived expertise	February 2025
Self‐care	August 2025/May 2025
Facilitating group sessions as lived experience workers	April 2025
Carer legal rights	Not covered^a^
Peer drift	July 2025/August 2025
Designated and declared roles	July 2025
Family carer discipline	April 2025/July 2025
Info on elder abuse and our duty of care	Not covered

^a^ This topic was addressed in a 2026 Educational Forum.

An interesting feature of the CoPs session was how CCV approached confidentiality. Confidentiality and privacy were documented in the CoP Report online document as ‘paramount’ features of the sessions to support ‘staff [to] feel safe to collaborate, share experiences and address issues’ and CCV engaged in direct conversation with the Connect centre workers to form an idiosyncratic agreement about confidentiality. Much like the Ways of Working documents discussed above, an agreement around confidentiality was formed by inviting staff to decide how their confidentiality, privacy, and safety would be protected within and beyond the sessions. The CoP Report Document describes the following confidentiality agreement. CCV facilitators and Connect centre workers agreed that:

- Discussions remain private
- Only agreed‐upon themes, gaps, and topics would be shared outside these spaces
- Shared information is intended to inform and drive change, support productivity, and escalate issues where necessary
- Any escalation would be discussed and handled in the interest of staff safety and service responsibility.

A flexible, collaborative approach to determine the bounds of confidentiality was shown to be used to contain these sessions and create safety within the CoPs.

## 4 Discussion

### 4.1 Power, Family Carers and CCV

Through this research, we became interested in understanding how power was acknowledged and addressed when family carers were given opportunities to lead and influence decision making. As the project continued, we grew increasingly interested in understanding what the redistribution of power looked like in a family carer‐led coordination body overseeing the service delivery of a peer‐led, community‐based service for mental health family carers.

Power became a significant focus in our research because navigating, addressing, and managing power dynamics remains central to the experiences of family carers and family carers workers for the well‐established reason that both globally and locally, family carers can lack power within mental health systems to influence better recovery outcomes for those they care for [11, 12, 13]. Powerlessness can manifest in many forms for family carers including a lack of access to vital information about their person coupled with power dynamics within services that elevate learned (often clinical) expertise over and above their lived expertise as family carers [10, 12, 17, p. 18; 30]. The less obvious reason for why power became the undercurrent in our research concerns the vision for a better mental health system heralded by the RCVMHS in our State of Victoria, Australia. In Volume 3 of the Final Report (2021) the Commission acknowledged that a core aim of the redesigned Victorian mental health system must involve ‘shifting [the] power’ embedded in the ‘professional and institutional hierarchies’ informing the mental health system and proactively redistribute and balance that power ‘to ensure that people with lived experience’ can influence, lead, and shape better futures [10, p. 21]. As a family carer lived experience led coordination body, CCV offers up a much‐needed case study for examining the reality of this vision to shift and share power.

In our research, we built upon findings in our companion paper which sought to systematically identify the core values embodied by CCV throughout their term coordinating the newly established Connect centres [15]. The four core values we identified were connection, responsiveness, consistency, and flexibility. In this paper, we spoke to three values: connection, consistency, and flexibility because our analysis of two additional projects, the online community co‐design project and the CoPs, demonstrated a continuation of these values and the extent to which power underpinned the enactment of each of them.

Our analysis demonstrated that a co‐design initiative and CoPs could not escape a discussion of power and how it was negotiated, addressed, and redistributed because both projects went beyond only inviting *participation* from Connect centre workers [33]. As we described in our introduction, co‐design and/or co‐production methods and CoPs are inherently linked with the address and negotiation of power; it was unsurprising that we witnessed power play out across both activities we analysed [20, 24, 33]. Rather, the online community co‐design and CoPs sought to redistribute power and invite collective decision making via a recognition that without proactively addressing power, neither project could affect good and relevant outcomes for Connect centre workers [33]. The actions CCV took in coordinating both projects rendered them directly and inextricably connected to power.

We showed how power sharing underscored connection, for example, as both a defining value and practice principle of Connect centre workers and was especially relevant to family carer lived experience leadership positions like CCV. We articulated this relationship by demonstrating how co‐design and co‐production methods served as tangible instances of connection (the value) at work because in the case of the online community co‐design project, staff were brought together to collaborate on the construction of a centralised online platform to support their day‐to‐day work with family carers. Our analysis of the online community co‐design process did not reveal groundbreaking insights into perfect co‐design and/or co‐production or a flawless coordination process led by CCV. Rather, our analysis showed a transparent, responsive, constrained process that leant on co‐production principles, especially acknowledging and addressing inequities of power, to co‐create an online community to improve ‘communication and collaboration between centres and with the support team of Connect Coordination Victoria (CCV).’ In addition, our analysis highlighted the direct link between connection as a coordinating value of CCV and the use of co‐design methods. The relationship between connection and co‐design efforts and methods for the FCLEW discipline warrants emphasising because it links the value of connection with a tangible practice or method (co‐design) that enacts this value [16]. For CCV, co‐design and co‐production methods enacted connection as a practice principle and central value because these methods brought people together with the goal to redistribute power.

Power also arose when discussing CCV's Ways of Working documents because through our analysis, these documents appeared to have a two‐fold role in grappling with power. The most notable way power was addressed by CCV through these agreements happened via an explicit acknowledgement of the need *for* Ways of Working documents—by virtue of including them in the first instance. Via the consistent use of Ways of Working documents, CCV disclosed their power often, while also sharing and distributing that power by inviting Connect centre workers to directly influence how they would work together. These agreements scaffolded the two CCV activities we analysed and invited upfront and consistent discussions of power. In this study, we also described how flexibility and power sharing related to each other by analysing CCV's approach to confidentiality within the CoPs. We found that CCV invited Connect centre staff to actively and meaningfully make decisions about what should be shared beyond the group and what conditions warranted sharing and escalation. It makes sense, for instance, that CCV would adopt a collaborative approach to confidentiality because family carers frequently report feeling shut out and excluded by mental health services who can perceive ‘the rights and needs of consumers’ in direct conflict with their needs and rights to general information about how best to support their person [17, p. 767]. Actively involving family carers in conversations about confidentiality and inviting them to have a say in where and how their information was shared and why, redistributed the power and accountability for protecting one another's safety and privacy within the CoPs across all group members (not just CCV). This flexible approach to power sharing, witnessed across the data we reported on in relation to the CCV‐led CoPs offers another example and further support for the unique value a family carer‐led coordinating body brings to the coordination of a tailored mental health family carer service.

### 4.2 Implications for Policy

As outlined in the introduction to this research, sharing power between those with learned expertise and those with lived experience is a principle at the heart of mental health system reform in Victoria following the RCVMHS. To ‘ensure that a future mental health and wellbeing system is founded on the needs and perspectives of those with lived experience […]’ the system needs to ‘proactively redistribute’ and balance that power so that system transformation can meet the needs of those who rely on that system to support them and those they care for [10, p. 19–20]. CCV's family carer leadership lends evidence for the reality of this power sharing vision of the RCVMHS while urging policymakers, wherever they may be around the world, to involve those with family carer lived experience in shaping, leading, and coordinating the design and delivery of mental health services and supports that directly impact them.

### 4.3 Implications for Practice

Others looking to coordinate tailored mental health services for family carers similar to the Connect centres both locally and globally could consider identifying and addressing power differences to avoid reinscribing harm to a cohort who have historically been on the receiving end of power imbalances that have marginalised their perspectives [11, 12, 21, 36, 37]. Arnstein's [33, p. 216] work on citizen participation is a vital resource and starting point for coordinators to grade and assess how power is distributed and to judge the degree of ‘real’ power family carers have in effecting decisions that directly impact them. As more tailored services for mental health family carers emerge globally, as we hope they will, practitioners could engage with research and resources that provide guidance on how to adopt inclusive and participatory approaches to working side‐by‐side with mental health family carers [9, 18, 20, 21, 22, 38, 39, 40, 41].

### 4.4 Limitations

This research was possible thanks to the meticulous record keeping of CCV's administration staff. The secondary data we used to inform our findings was not a primary source of data, however. We found that reporting on secondary data required an ardent commitment to transparency. Researchers looking to adopt a similar method could benefit from methodically noting which findings arise from which documents and making these connections very explicit for readers in their outputs. In future research, we look forward to firming up our findings by including primary sources of data through fieldwork and interviews. This future field work with mental health family carers will further strengthen the patient and public involvement of our family carer‐led research.

## 5 Conclusion

Across two research publications we emphasised the importance of family carer lived experience coordinators leading tailored services for family carers to systematically identify their practice values by looking first to what they do and how they do it [15. We took an *actions first* approach to this research because mental health system reform, locally and globally, requires action: action underpinned by the kinds of values and lived experience advocacy and leadership, as described here, that generates services that directly address the needs of those most underserved by our mental health systems. Family carer coordinators like CCV, choose to intentionally employ their lived experience perspective to the benefit of others. They are therefore uniquely situated and especially motivated to acknowledge and address power differences because they understand via their lived experience what it is to lack power to affect better futures for themselves and those they care for.

## Author Contributions

**Jessica Phillips:** conceptualisation, investigation, methodology, formal analysis, writing – original draft, writing – review and editing, data curation, project management, project administration. **Lisa Casaceli:** writing – original draft, review and editing, validation. **Clare Fleming:** writing – original draft, review and editing, validation, project administration. **Jennifer Bite:** conceptualisation, writing – original draft, review and editing. **Caroline Walters:** writing – original draft, review and editing, validation, mentoring. **Joanna Tilkeridis:** project supervision, writing – original draft, review and editing, validation, project administration. **Marie Piu:** conceptualisation, validation, project supervision, writing – review and editing. **Melissa Petrakis:** conceptualisation, methodology, formal analysis, writing – original draft review and editing, project supervision, and mentoring.

## Conflicts of Interest

J. B. left her role at Tandem part‐way through the research project to commence work at the Victorian Department of Health. The views expressed by J. B. here reflect her work with CCV and Tandem, and not the Department of Health. The other authors declare no conflicts of interest.

## Data Availability

The data that support the findings of this study are available from the corresponding author upon reasonable request.
